# Radical prostatectomy in the treatment of prostate cancer. The experience of the Urology Clinic of „Prof. Dr. Th. Burghele” Clinical Hospital

**Published:** 2009

**Authors:** V Ambert, B Braticevici, D Damian, I Chira, V Iconaru, T Radu, T Constantin

**Affiliations:** * „Prof. Dr. Th. Burghele” Clinical Hospital

**Keywords:** Prostate cancer, Prostatic Specific Antigen, Trans Rectal Ultrasound with guided punction, multiple randomised punctions or extended punction, Gleason score, radical prostatectomy, epidural catheter, Santorini plexus, neurovascular bundles, retrograde cystography, urinary incontinence, erectile dysfunction

## Abstract

**Introduction**: radical prostatectomy remains a real challenge for most of the urologists.

Our study’s objective is bringing into discussion the main aspects related to the technique we use, the intra and post-operatory complications, as well as the short-term analysis of the results in PC treatment using RP in the „Prof. Dr. Th. Burghele” Clinical Hospital - Clinic of Urology.

**Material and methods:** between 1999 and 2007, 59 patients with PC, aged 48-74, were operated in our clinic.

We began to recommend prostate biopsy (PB) to all patients with PSA higher than 4 ng/ml and, in the last years, higher than 2,5 ng/ml.

A change in our attitude is related to the PB. At the beginning, we tried to perform „targeted” punctions, ultrasound guided especially in suspect zones, afterwards, we performed randomised punctions at all the patients, no matter the aspect of TRUS and we have increased the number of punctions accordingly to the prostate volume (minimum 6 punctions, maximum 12). The most used was the 10 core punction.

The T classification, according to the clinical diagnosis, of the 59 operated patients: **T1** cT1 a-b - 4 cases; cT1 c - 39 cases;**T2** cT2 a - 12 cases; cT2 b - c - 4 cases.

The RP surgical technique was the classic one, described by P.C. Walsh – the first surgical step, in all cases was lymphadenectomy.

**Results:** of all the patients that went through RP, 56 cases are still in our records.

We can consider healed 24 patients with PC, followed for 3 years post-surgery, because they had no need of therapy and the PSA is maintained below 0.02 ng / ml.

The Gleason score - between the pre-operatory established diagnosis by punction and the anatomic-pathological examination of the piece, there were some differences: the concordance was in 48% of the cases; in 39% of the patients, the biopsy specimen had a lower Gleason score than the surgery specimen, and in 13 % a higher score, the most common error was caused by sampling.

The correlation between the pre-operatory evaluated clinical stage and the pathological clinical stage was of 57%.

The most important late postoperative complications of RP were: urinary incontinence and erectile dysfunction.

In our study, we have recorded late postoperative: complete urinary incontinence in 4 cases (6.7%), erection was maintained after bilateral preservation of neurovascular bandelets in 90% of cases and after unilateral preservation in 71% of cases.

Due to the short following period, we can’t say if the operated patients by us had a benefit regarding the general surviving period;

The personalized interpretation of the increase of serum PSA levels after surgery represents a possible problem regarding the indication of complementary treatment.

## Introduction

At the beginning of the eighth decade of the past century, the introduction of serum PSA in the usual medical practice has represented a major change in diagnosis and treatment of prostate cancer (PC). Due to its discovery in early stages, the number of curatively treated PC cases has risen significantly and for this particular reason, the treatment methods have also diversified continuously. The new radical treatment methods (brachitherapy, cryotherapy, HIFU etc.), completing the classical ones (external radiotherapy and radical prostatectomy), have been developed with the intention of offering PC patients a treatment as radical as possible (the absence of positive margins) with minimum per-operatory complications, ensuring the complete recovery of potency and continence and also taking into account the life expectancy.

Once the indication for radical treatment has been established, the urologist must choose between the „sufficient” extirpation of periprostatic tissues and the preservation of the integrity of neurovascular structure, which assures the normal urinary, intestinal, and sexual functions. In many cases, the accomplishment of one objective implies the deliberate renunciation to the other one.

Although at present there are more possibilities for radical treatment of PC in Romania, the radical prostatectomy remains a real challenge for most of the urologists.

The objective of our study is to bring into discussion the main aspects related to the used technique, the intra and postoperative complications, as well as the short-term (< 5 years) analysis of the results in PC treatment using RP in the „Prof. Dr. Th. Burghele” Clinical Hospital - Clinic of Urology.

## Material and methods

Between 1999 and 2007, 59 patients with PC (aged 48-74) were operated in our clinic. In 2008, 23 more PC cases were operated in our clinic, but these were not included in our study. The characteristics of the studied group are presented in **Table no. 1**:

**Table 1:** Characteristics of the studied group

Characteristics	Patients with PC treated by RP
No.	59
Mean age	61.5 years old
PSA	3.5 - 23.5 ng/ml
Prostate volume	34 - 69 cc
Number of biopsies	6 – 10 / patient

**Fig. 1:**
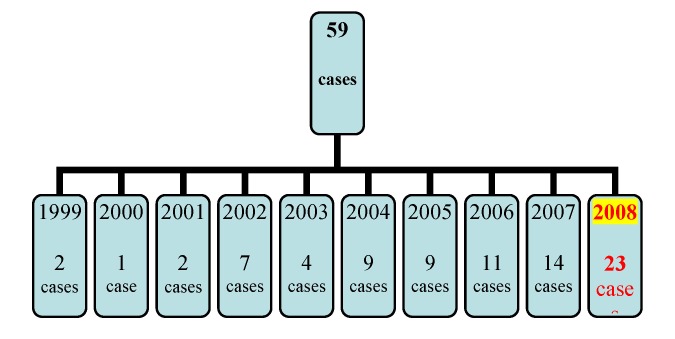
Number of the PC cases operated on annually, 1999-2007

The number of cases operated on each year, 1999 – 2007, is presented in **Fig. 1**.

As one can observe, the number of operated cases increased every year. This variation in the number of cases operated on each year could be explained by the absence of a correct methodology in order to establish the diagnosis of PC.

Although we have had access to transrectal ultrasound guided prostate biopsy since 1996 and to serum PSA determination since 1994, the number of cases in which the puncture was indicated was very small, even if the serum PSA was higher than the normal value (< 4ng/mL). When the digital rectal examination (DRE) didn’t show any PC suspicion, the patients were punctured in most cases, if the serum PSA was higher than 15-20ng/mL, these values being considered „significant” in order to establish the diagnosis.

Although we were having access to a new and efficient method of PC diagnosis in early stages (serum PSA), we did not either trust or use it correctly. After achieving more and more experience and starting to notice that there were many cases in which operated patients with serum PSA, had levels higher than 15ng/mL and also presented positive margins, we began to recommend prostate biopsy (PB) to all patients with PSA higher than 4ng/mL or, in the last years, higher than 2.5ng/mL.

Another change in our attitude is related to the PB. At the beginning, we tried to perform „targeted” punctures, ultrasound guided especially in hypoechoic and/or asymmetric zones, which we considered to be characterized by a high probability of PC. In those situations, if we could not detect those zones at TRUS, the number of PC remained small or the patients remained unpunctured. In time, our growing experience determined us to perform randomized punctures at all the „suspect” patients, no matter the aspect of TRUS. We also increased the number of punctures in accordance to the prostate volume (minimum 6 punctures, maximum 12).

By having done this, we registered a significant increase of diagnosed PC number, most of them in curable stages. 

Our protocol diagnosis includes: the evaluation of lower urinary tract symptoms (LUTS) using IPSS, digital rectal examination (DRE), the total/free serum PSA report and, in „suspect” cases, TRUS examination with multiple randomized punctures.

The clinical symptomatology in the study group, quantified by I-PSS score, was present * in 86,5% of operated cases in our clinic, as presented below:

Minimum symptomatology: 10 cases

Medium symptomatology: 29 cases

Severe symptomatology: 12 cases

* no symptomatology : 8 cases

**Fig. 2:**
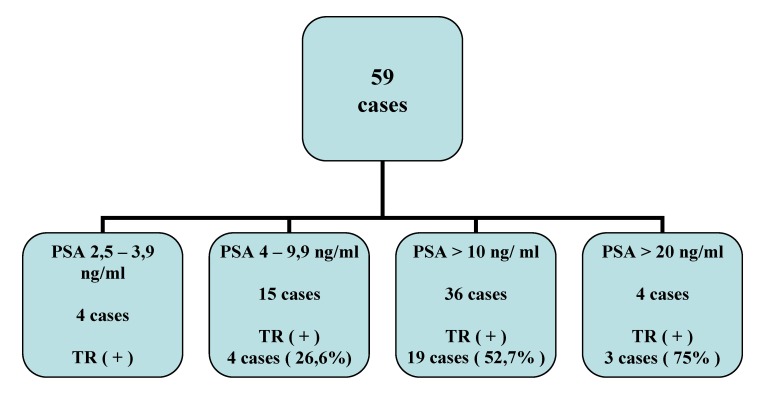
The relation between serum PSA and the PC suspicion at DRE

DRE was performed in all patients, 17 cases (28,8 %) being considered „suspect”. We present the correspondence between DRE and serum PSA in all 59 patients with radical prostatectomy (**Fig. 2**):

Serum PSA values represented a key element in diagnosis establishment. We arbitrarily decreased the maximum level of normal PSA, because the percentage of PC detected at levels between 3 and 4ng/mL is, according to literature data, 26.9%, most of the PCs being clinically significant (8). We wanted to see if this decrease could bring benefits in PC diagnosis.

In our study, we have had 18 patients with serum PSA values between 2.5 and 3,9ng/mL, whilst DRE was normal. In 4 of these patients (22,2 %) the puncture detected PC. If the limit value of PSA would not have been lowered, the puncture indication would not exist anymore and the patients would not have been punctured. The four patients were included in the group with sextant biopsy. All these patients had a prostate volume lower than 60 cc. The Gleason score was 6 in 2 cases, respectively 7 in the other 2.

Only 14 patients (23.7%) of those operated for PC, were punctured for higher levels of serum PSA accidentally detected on usual check-up. This is a very small number, considering that the PSA is not an obligatory test in the Romanian health insurance system.

**Fig. 3:**
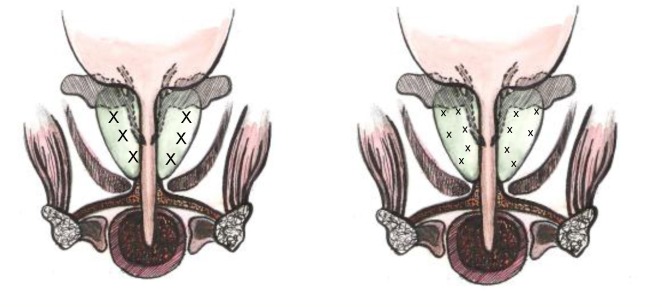
Randomized sextant and extended puncture

Once the biopsy indication has been established, we used exclusively TRUS randomized punctures. The exceptions were represented by patients with levels of serum PSA higher than 20 ng/mL and with high consistency zones, PC suspect at DRE. Most of these patients were given direct, multiple, digitally guided puncture of the „suspect” lobes. Normally, we performed 6 punctures minimum, according to the Hogde method and 12 punctures maximum, all in the peripheral zone of the gland (**Fig. 3**):

The mostly used was the 10-core puncture.

The prostate volume, determined before the puncture using TRUS is very important for the number of biopsies. Our experience shows that a biopsy protocol which includes more than 8 punctures is useful only for prostates with volumes higher than 30 cc.

After the PC diagnosis, we tried to establish the stage of the disease.

The clinical stage was established using the TNM classification from 2002.

For the clinical staging, we used PB, pulmonary Rx, liver ultrasound examination, and CT scan besides DRE and serum PA.

**Fig. 5:**
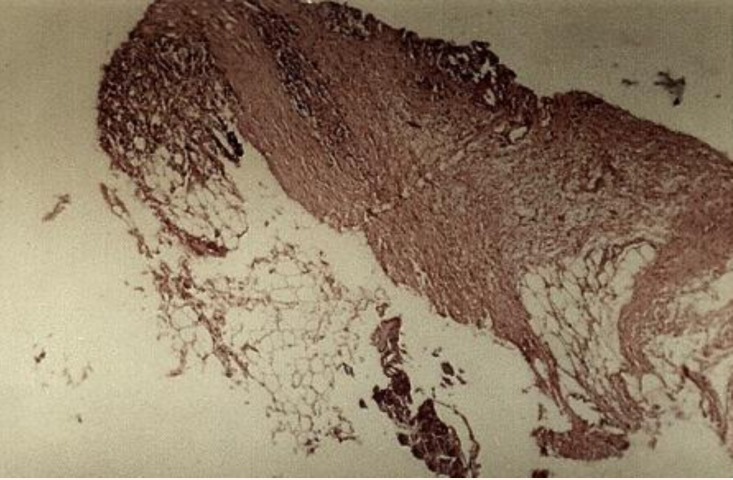
BP – in the proximity of the carcinoma area the following can be visualized: the prostate capsule and the periprostatic fat which are not tumor invaded

If correctly performed, the PB can contribute to the clinical staging of PC (**Fig. 5**):

**Fig. 6:**
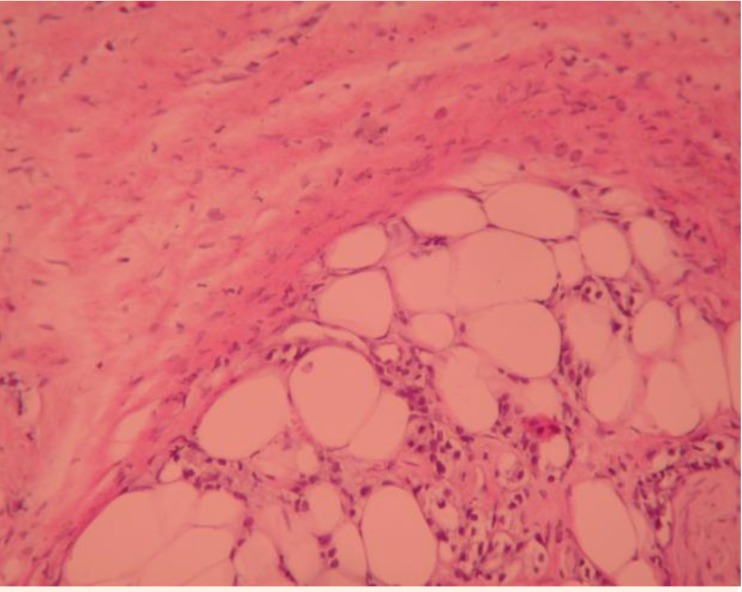
BP the prostate capsule and the periprostatic fat, which are tumorly invaded

**Fig. 7:**
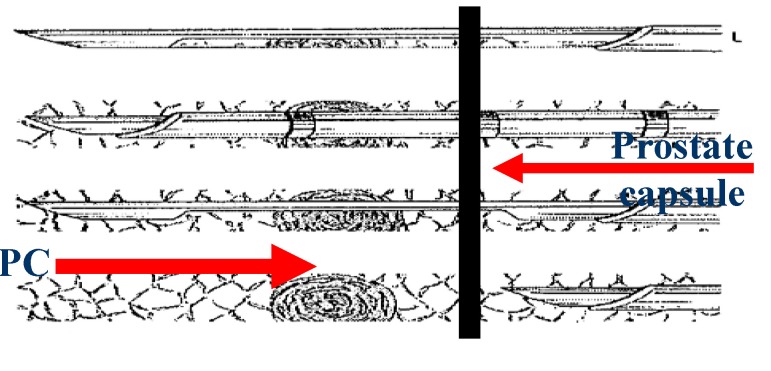
The correct needle placement for biopsy

Technically, before engaging the automatic puncture system, the needle must be placed so that it does not depress the prostate capsule. In this position, it will gather periprostatic fat, capsule, normal and pathologic prostatic tissue (**Fig. 7**).

The T classification, according to the clinical diagnosis, of the 59 operated patients (**Table 1**):

**Table 1:** The stage classification of the 59 PC cases

**T1** cT1 a-b - 4 cases; cT1 c - 39 cases	**T2** cT2 a - 12 cases; cT2 b - c - 4 cases

**Fig. 8:**
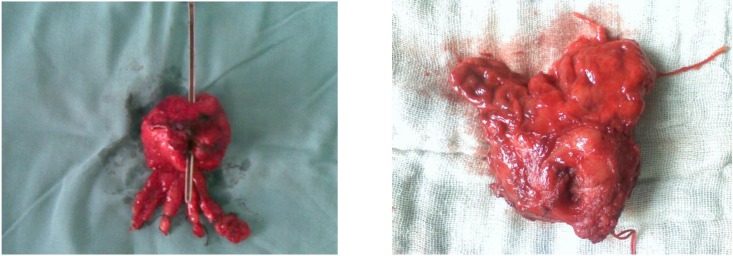
Surgery piece – radical prostatectomy. A – No positive margins B – Positive margins (left lobe tumor infiltration: seminal vesicle and the apex)

P.C. Walsh described the RP surgical technique to be the classic one. Lymphadenectomy was performed in all cases as the first surgery step. In patients who had indication of RP, we practiced bilateral lymphadenectomy, limited to obturatory fossa, because we considered that all patients were in a clinically localized stage. In some particular cases, when Gleason score was higher than 7, with more than 3 positive bioptic fragments recorded, and the PSA was higher than 15ng/mL, we expanded lymphadenectomy beyond obturatory lymph nodes. In patients clinically staged < cT2a, with PSA level less than 10ng/mL, Gleason < 7 and less than 3 positive fragments of bioptic specimens, we practiced lymphadenectomy followed by RP even if macroscopic and palpable examination of the ggl. suggested neoplasic invasion. In 97% of these cases, the HP (histopatological) exam indicated lymphadenitis (inflammatory disease). After surgery, none of these patients (6 cases) with ggl invasion, presented any positive margins on the prostate specimen. After three years post procedure, none of these patients had local or serum PSA recurrence (**Fig. 8**).

We tried to spare neurovascular bundles whenever possible. When technically possible, we preserved the neurovascular bundles unilaterally or bilaterally, regardless the age, intraoperative. The surgical technique that we have used consisted in the incision of the visceral lamina of endopelvic fascia on the side of the prostate, followed by the dissection of preprostatic fascia as anterior as possible, to allow lateral mobilization of the neurovascular bundles and avoid the use of electrocauter. 

In 7 cases, neurovascular bundles were damaged due to haemostatic maneuvers.

We tried to preserve the bladder’s neck as much as possible and we modeled it in a „rocket” shape.

The anastomosis of the bladder’s neck was sutured through 5 – 6 separate vicryl 000 stitches to the membranous urethra.

The intra-operative complications that we faced were the following: intra-operative bleeding was between 150 - 1500mL requiring blood transfusion intra- and post operative - 1-4 units of entire blood. In 4 cases, intra-operative bleeding was between 1500 -2100mL.

Bleeding was abundant in 18 cases. In all cases, bleeding was stopped, but the haemostatic maneuvers extended the surgery time and the sectioning of the urethra was difficult, creating postoperative continence problems.

In 10 cases, bleeding occurred due to the lack of haemostatic control of the preprostatic venous plexus and in other 8 cases, the bleeding appeared due to neurovascular bundles injury. In those cases, bleeding control imposed the incision between the ligatures of the neurovascular bundles unilaterally in 4 cases and bilaterally in one case.

In most cases, the preprostatic venous plexus has been sutured with slow absorbable braided multifilament material, passed through with a curved needle, and sometimes has been attached to the periosteum of pubic symphysis. In our experience, we found this maneuver to be more appropriate than the passage of a „L” shaped forceps behind the venous plexus, in order to catch the thread of haemostasis. In all cases, we practiced the ligation of the veins on the front of the prostate with a nylon thread, so as to prevent venous tide.

In order to reduce the bleeding, we used the following techniques:

- positioned the lower limbs of the patient in the lowest reachable manner plus Trendelenburg emphasized;

- the first option for anesthesia technique was epidural catheter, this was injected with ropivacain in concentration of 1%, about 15-20mL, which provided 4 hours of surgical anesthesia (with muscle relaxation). A chemical sympathectomy that reduced bleeding due the vasoconstriction was also achieved during surgery. After surgery this catheter was kept in place, offering the ability to maintain (by repeated injections of 4-6mL/h ropivacain of 0.2%) a very good analgesia during the first 48 hours, patients’ recovery being spectacular;

- decreasing I.V. hydration (500mL/h).

In order to verify the quality of anastomosis, intraoperatory, we practiced a tightness test to all patients (200cc were injected through the urethral catheter). If the test was good (just a small amount of liquid leaks) we introduced 20cc in the balloon of Foley catheter, and set a continuous lavage for 48 hours.

In all cases, we have left a drain tube, lateroanastomosis, released by a small flank incision. At first, the time for the surgery was set at 4 hours and it decreased progressively at 2 hours. In one case, in the first 12 post operatory hours, the patient accused chest pain and EKG recorded acute myocardial ischemia followed in a few hours by death.

Mobilizing patients was early in the first 24 - 48 hours and, on the third post-surgery day they began Kahler exercises.

**Fig. 9:**
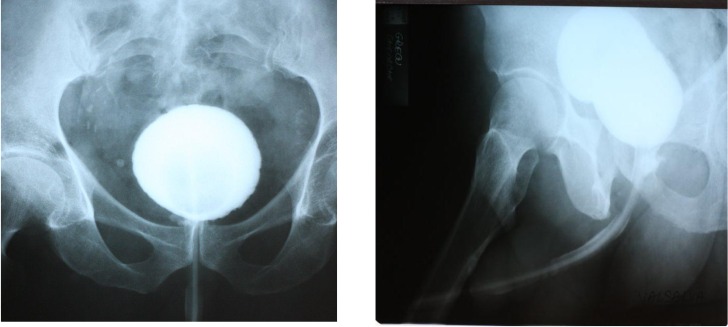
Retrograde cystography, at day 6 postoperative, showing a small posterior continuity solution at urethral-bladder anastomosis.

Between day 5 and day 7, a retrograde cystography, on the urethral catheter, was performed with an antero-posterior and lateral exposure, in order to assess the tightness of the anastomosis. If cystography showed no loss, the urethral catheter was removed in the same day or the next day (**Fig. 9**):

We have not used adjuvant hormonal treatment for the 4 patients with RP and values > 20ng/mL of the serum PSA. 

## Results

Of all the patients who had undergone through RP, 56 cases are still in our records. We estimate that, from a total of 31 patients, followed by 3 post-surgical years, 24 patients with PC can be considered healed because they had no need for therapy and the PSA was maintained below 0.02ng/mL.

For 25 patients with CP, operated through RP, we do not have a 3-year follow-up period. We estimate that the rate of patients considered to be healed will be higher in this batch, because the selection for the intervention was more rigorous. We have registered a number of 34 post surgical early complications. Of these, the most frequent were: prolonged drainage (over 6 days) of the urine drainage tube - 3 cases, wound suppuration - 1 case, inosculate dehiscence - 1 case, severe LUTS phenomena, accompanied by macroscopic hematurya (caused by the suture material) - 1 case, febrile state - 1 case.

Anastomoses’ dehiscence was partial (posterior half) and resolved through surgical reintervention at 16 days and the patient was continent at the end. We recorded a total of 28 cases of urinary incontinence at the urethral catheter’s removal. Of these, in 18 cases, continence was totally restored within 45 days. After 90 days, the other 5 patients regained full continence.

Of all the patients who had loss of urine postoperative, the majority accused this loss when changing the position, with the emergence of urination sensation (until the patient got to the bathroom), or spontaneously in orthostatic position. The amount of lost urine ranged from a few drops to 15-30mL. In order to correct incontinence, patients have used absorbent pads.

The most important late postoperative complications of RP were urinary incontinence and erectile dysfunction.

2 years postoperative, at a routine check-up, we recorded 1 case with a remaining foreign body (textiloma), in the lateral side of the bladder. Due to the fact that it appeared to be situated near the iliac vessel, we thought that it might have been an adenopathy. The patient was operated and the remaining body was extracted without any complications. The patient is continent and maintained potency.

Late postoperative problems were recorded: urinary incontinence in 4 cases (6.7%) and 1 case with stricture of anterior urethra. 

Urine incontinence appeared and manifested itself by heavy losses, uncontrolled urine, both in orthostatic and decubitus position. Those patients’ attempts to perform Kahler exercises were unsuccessful (patients have noted the connection between the intention to contract sphincter and the lack of this sensation). 

Sphincter’s injury is a severe complication, and the total incontinence that comes from this is a disability, which is extremely hard to be accepted by the patients.

In our statistics, we noticed that most often sphincter’s injury was related to haemostatic problems in the pre-prostatic venous plexus. The ligature of Santorini plexus is one of the most important points of intervention and once the haemostasis is achieved, this allows us to have a good visibility in the operating field during the urethral wall incision. In patients who have been bleeding more than 300mL of venous preprostatic plexus or those whose local excision required a broader apex, there were more important continence problems compared to the ones of the other patients. 

In one case, we tried to correct urinary incontinence occurring postoperative PR by repeated injections of collagen (5 sessions), without any results. Finally, we performed a surgical intervention setting a polypropylene bundle attached to the descending branches of the pubis. This method also failed to reduce significantly the loss of urine.

Erectile dysfunction in patients with CP has not been preoperatively evaluated with a score. The factors affecting erectile function in our statistics were:

- Patients' age (**Table 2**): postoperative recovery of erectile function was inversely proportional to patients’ age.

**Table 2:** The relation between PC patients’ age and the postoperative erectile dysfunction

Age (years)	% postoperative potency
50-59	82%
60-69	58%
>70	12%

- The time since the intervention has influenced the recovery of sexual function (**Table 3**). Complete recovery of erectile function was slow and required 18 months or more. The recovery of erectile function required more time than that of the continence.

**Table 3:** **The connection between the time from intervention and the degree of sexual function recovery**. * Patients aged between 50 - 60 years

Postoperative period (months)	% Potency *
3	28%
6	48%
12	67%
18	82%

- The comorbidities and the medication administered for these (diabetes, hypertension, and arrhythmia) significantly influenced the potency in a negative way. Among our patients, those who smoked more than a pack of cigarettes per day and were aged over 65 had erectile dysfunction in 92% of cases.

-Prostate volume did not influence the postoperative maintaining of potency. Men who were potent preoperatively and used phosphodiestherasis inhibitors (PDE-5) had efficient erections postoperatively. An erection was maintained after bilateral preservation of PR (PR pb) of bandelets in 90% of cases, and after PR pb alone in 71% of cases. Patients who have responded best to medication with inhibitors of PDE-5 were those aged ≤ 60 years old, with bilaterally maintained bandelets and / or those with spontaneous erectile function, which existed preoperatively.

In patients with intraoperative wide excisions at the apex level and / or at the prostate base and in those with postoperative positive margins, the erectile dysfunction was present in 28% of the cases higher than in other patients. 

One single death was recorded postoperatively (within 24 hours postoperatively due to an acute myocardial ischemic accident).

We performed RP in 4 PC cases in which the diagnosis was established by the trans-urethral resection of the prostate (TUR-P). In the other 3 cases we considered that, following TUR-P, the RP was technically impossible because of the „adherence” process taking place between the anterior part of the prostate and the pubic symphysis, particularly due to the control problems of subpubian venous plexus. Those cases were sent to Rx therapy and, concomitantly, hormonal therapy with LH-RH analogues. 

The correlation between the preoperative evaluated clinical stage and the pathological clinical stage was of 57%. 

The non-correlation between the differentiation degree of the PB and the surgery specimens is well known. The Gleason score of the specimen of the surgery is unanimously accepted as the real one for the patients who were surgically treated for PC. The most common non-correlations are caused by sampling errors, the pathologist experience, border cases, etc.

Regarding the correlation (the correspondence between the tumor differentiation degree and extension) between the diagnoses preoperatively established by puncture and the anatomic-pathological examination of the piece, there were some differences, as follows: 

- For Gleason score – the concordance was in 48% of the cases; in 39% of the patients, the biopsy specimen had a lower Gleason score than the surgery specimen and in 13 % a higher score. In our statistic, the most common error was caused by sampling.

- The extension of the tumor’s degree was concordant in 42% of the cases when only one bioptic fragment was positive, in 25 cases.

In 34 cases the PC diagnosis has been confirmed on more than one prostatic bioptic fragment; in this situation the concordance was of 57%.

The histological exam of lymph node groups has emphasized local metastases in 6 cases.

RP patients were postoperatively monitored according to the following protocol: TR, PSA level and echographic examination with junction and residue check at 3, 6, 9 and 12 months during the first year and at every 12 months during the first 3 years. The threshold of PSA levels from which we considered that patient required additional investigations was set at > 0.2ng/mL.

In this setting, we noticed a significant decrease of PSA level in 6 patients immediately after the operation, but not under 0.02ng/mL. In time, early postoperatively, we noticed an increase of PSA levels in these patients. We considered that all those patients have been under-staged and there were no relapses. Knowing the relation between early biochemical progression and the higher risk of metastasis, which influences the specific mortality, we initiated the therapy with LH-RH analogues and antiandrogens for all these patients.

In only one case of the 31 for which we have a 3-year follow-up period, we recorded a relapse after 1 and a half year from intervention. The relapse was suggested by the progressive increase of PSA levels reaching 2.5ng/mL.

In this case, we examined the patient by ETR using Color Power Doppler and CT. The ETR did not show anything pathological in the bladder-urethral junction area and so, this area was not punctured. The patient has been treated with anti-androgens and LH-RH analogues and has been continuously monitored clinically and paraclinically. 

If we should discuss the advantages and the disadvantages of RP in the treatment of localized PC, first of all we should ask the following question: Does this technique offer benefits, for a significant number of patients, regarding the prevention and the progression of the disease?

The things we can affirm are:

-Due to the short following period, we cannot say if the patients operated in our hospital benefited from the general surviving period.

-The personal interpretation of the increase of serum PSA levels after surgery represents a possible problem regarding the indication of complementary treatment.

## Conclusions

From our experience, we can affirm that, the risk-benefits ratio for every patient must be in the favor of the last, without unnecessary exposure to the first.

In the final evaluation of each patient’s prognostic, the HP exam of the surgical piece is very important. For that particular reason, an experienced pathologist must perform this exam. The difficult (uncertain) cases should be discussed by an urologist and a pathologist, avoiding the supplementary unnecessary treatments or their absence in patients who need it.
